# Porphyrins as Catalysts in Scalable Organic Reactions

**DOI:** 10.3390/molecules21030310

**Published:** 2016-03-08

**Authors:** Juan C. Barona-Castaño, Christian C. Carmona-Vargas, Timothy J. Brocksom, Kleber T. de Oliveira

**Affiliations:** Departamento de Química, Universidade Federal de São Carlos, São Carlos 13565-905, Brazil; barona.castano.juanc@gmail.com (J.C.B.-C.); ccamiloc30@gmail.com (C.C.C.-V.); brocksom@terra.com.br (T.J.B.)

**Keywords:** porphyrins, catalysis, scalable reactions, sensitizers, continuous flow photocatalysis

## Abstract

Catalysis is a topic of continuous interest since it was discovered in chemistry centuries ago. Aiming at the advance of reactions for efficient processes, a number of approaches have been developed over the last 180 years, and more recently, porphyrins occupy an important role in this field. Porphyrins and metalloporphyrins are fascinating compounds which are involved in a number of synthetic transformations of great interest for industry and academy. The aim of this review is to cover the most recent progress in reactions catalysed by porphyrins in scalable procedures, thus presenting the state of the art in reactions of epoxidation, sulfoxidation, oxidation of alcohols to carbonyl compounds and C–H functionalization. In addition, the use of porphyrins as photocatalysts in continuous flow processes is covered.

## 1. A Brief History of Catalysis

Catalysis is a phenomenon observed since ancient times when Valerius Cordus (1514–1544) converted ethanol to ethyl ether using sulfuric acid as catalyst [1]. Formally, this term was proposed in 1835 by Berzelius (1779–1848), who defined catalysis as the ability of substances “to awaken affinities, which are asleep at a particular temperature, by their mere presence and not by their own affinity” [1,2]. Later, Ostwald (Nobel Prize in Chemistry in 1909) introduced a rational physical chemical definition of a catalyst in 1895 as a “substance that can change the reaction rate (accelerate or inhibit) without modification of the energy factors of the reaction” [2]. However, the complete molecular basis of the catalytic processes was established just one century later, [3] and now is widely applied on a large scale. One important and historical example in industry is the Haber–Bosch process for ammonia synthesis [3].

In fact, acid-base and metal catalysis have been the most recurrent procedures in industry and academy over the last centuries, allowing the growth of chemistry with many benefits for society. However, very complex systems have been developed more recently, including mixed metallo-organic materials and multiple catalysts [4].

Currently, almost all the chemical processes in industry are mediated by catalysts implying large annual expenses, involving green and energy-saving processes. In 2009 Noyori [5] stated “I personally consider that catalysis is the most important subject in chemistry and also technology—80 per cent of all commercial products are made by catalysis and the total market of these commercial products is $7 trillion [£4.3 trillion]”; in a very recent editorial Zhou affirms that it is now 90% [6].

Taking into account the challenges of the global economy and the needs for more sustainable chemical processes, catalysed reactions represent an important role for the development of new synthetic methods to generate target molecules in fewer steps and with less chemical waste. Certainly, the design, synthesis and applications of new catalysts are a hot research topic in the three scientific disciplines, considered essential for catalysis: chemical engineering, inorganic chemistry and organic chemistry.

In this context, it is important to highlight that porphyrin derivatives are special molecular scaffolds, which present relevant activities and cost-competitive transformations in these fields, thus deserving attention. In this review, we intend to cover the most relevant scalable porphyrin-catalysed procedures, showing how these compounds represent broad applications in chemistry.

## 2. General Concepts in Porphyrin Catalysis

**Figure 1:**
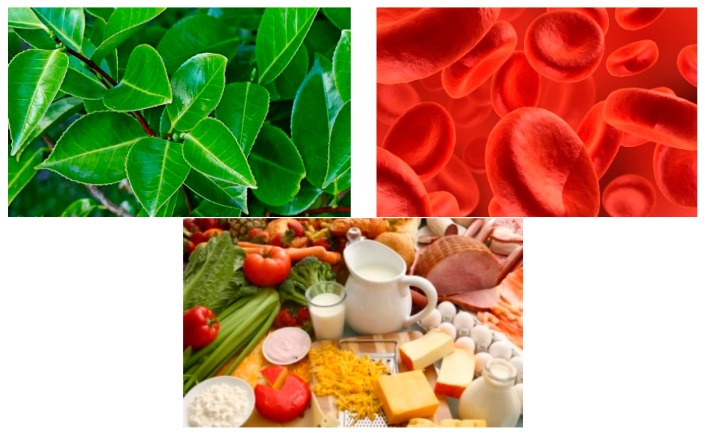
Porphyrin-derived pigments that participate in life processes.

**Figure 2:**
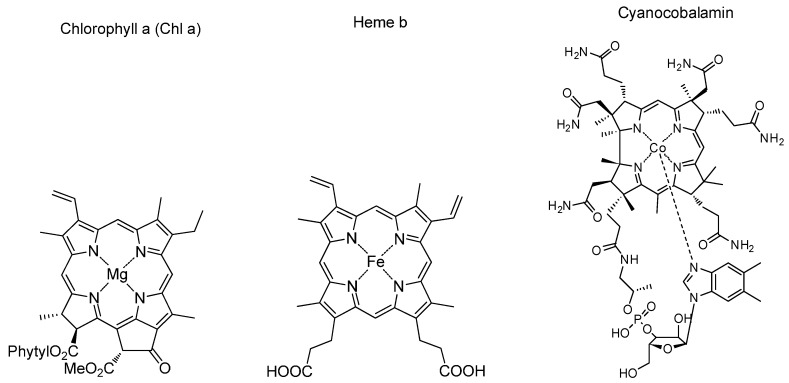
Metalloporphyrin derivatives involved in life processes.

Porphyrins and their derivatives are a class of naturally occurring macrocyclic compounds with intense colour, that have been extensively studied [7,8,9] due to the key role played in some life processes (Figure 1 and Figure 2) and involving their special aromatic structure (18π electrons). A suitable example that can be given is the heme group which contains an iron-porphyrin complex which coordinates oxygen and carbon dioxide for cellular carriage [10], responsible for cellular respiration, and contributing to the catalytic activities of many enzymes as cofactors [11]. This is one of the reasons why porphyrin-derived pigments are called “the Pigments of Life” (Figure 1 and Figure 2) [12,13].

The history of the study of metalloporphyrins’ activities began in 1747 when Menghini demonstrated for the first time the presence of iron in blood [14]. Hoppe and Seyler in 1871 isolated porphyrins from blood, describing these compounds as pyrrole derivatives, and in 1940 the structure and biological functions of iron porphyrin complexes were well established [15]. Nowadays, this kind of metalloporphyrins is well known since they are prosthetic group of an important class of proteins and enzymes called hemoproteins.

Metalloporphyrins have also been widely studied as bioinspired models of cytochrome P-450 (hemoproteins), and have exhibited catalytic activity for highly selective monooxygenation reactions [16], which proceed via formation of a high valence metal-oxygen complex intermediate. In 1979, Groves and co–workers developed the first oxidation system using a synthetic metalloporphyrin as a bioinspired catalyst [17]. 

**Scheme 1:**
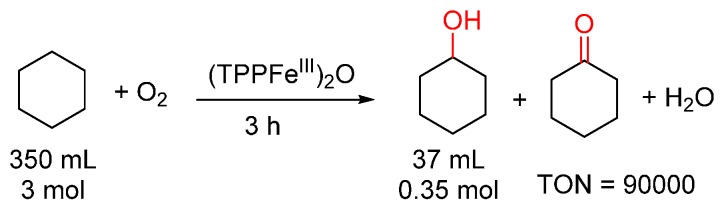
Bioinspired aerobic C–H oxidation of cyclohexane by a *μ*-oxo-iron porphyrin.

Since then, the synthesis and study of these very robust macrocycles inspired by biological systems, have been incorporated into the development of sophisticated bulky, chiral, and surface linked catalysts, because of a variety of properties that turn them useful for organic synthesis (Scheme 1) [18].

**Figure 3:**
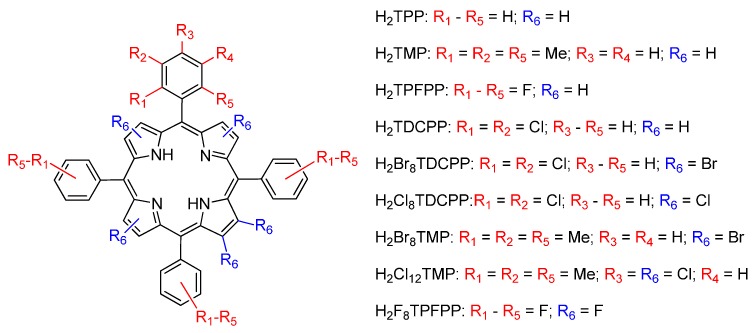
Common synthetic β- and *meso-*substituted porphyrins.

The development of metalloporphyrin catalysts as synthetic tools has recently evolved significantly [19]. For instance, in catalytic C–H oxidation reactions, the simplest metalloporphyrins without substituents at the *meso* positions are not useful because they undergo fast oxidative degradation [20]. The degradation process (the catalase reaction) leads to hydroxylation at the *meso* position, followed by other processes that occur in natural *heme* degradation, in order to form *meso*-hydroxyporphyrin derivatives which then inactivate the catalytic activity [20]. Therefore, it has been demonstrated that the introduction of phenyl or related groups at the *meso* positions (Figure 3) is a good strategy which provides efficient catalytic oxidations by protecting these reactive sites [19].

**Figure 4:**
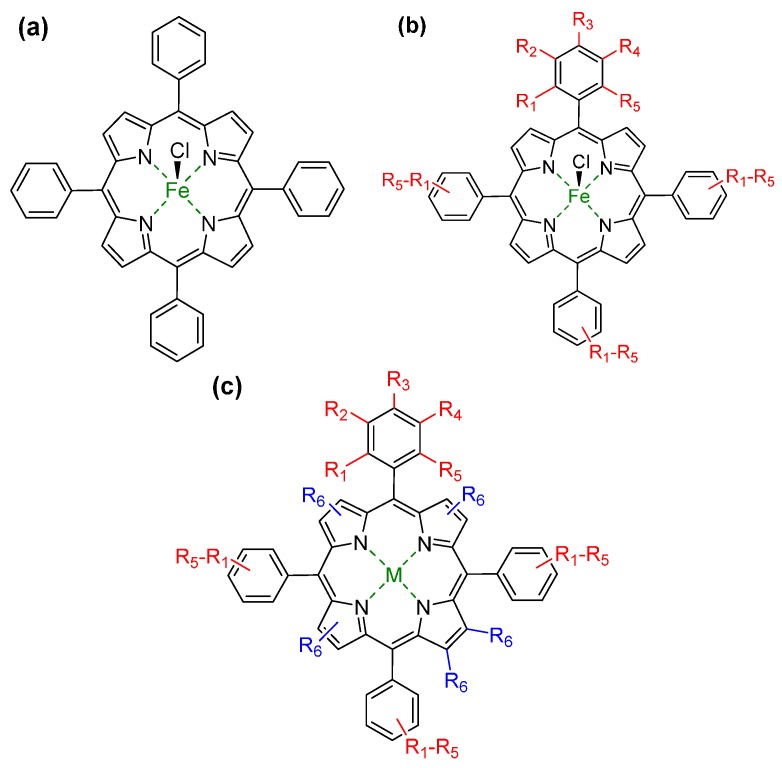
Classification of metalloporphyrin catalysts. (**a**) First generation; (**b**) Second generation; (**c**) Third generation. R_1_–R_5_ represent EWG or bulky groups and R_6_ halogens.

In 1997, Dolphin and Traylor proposed a classification system for all the different metalloporphyrins used in catalysis based on their structural features (Figure 4) [21]. These authors classified the synthetic metalloporphyrins without substituents in the aryl moiety at the *meso* positions as first generation porphyrins, and one example is the *meso*-tetraphenylporphyrin iron(III) chloride ([Fe(TPP)]Cl) that Groves *et al.* employed in cytochrome P-450 bioinspired catalysis (Figure 4a) [17].

Nonetheless, *meso*–phenyl-substituted metalloporphyrins bearing electronegative and bulky groups were classified as second-generation porphyrins such as *meso*-tetrakis(pentafluorophenyl)porphyrin iron(III) chloride and *meso*-tetramesitylporphyrin iron(III) chloride (TMPFeCl) (Figure 4b). The introduction of electron-withdrawing groups (such as halogens) in the β-pyrrole positions of second-generation porphyrins yields the so-called third generation porphyrin derivatives (Figure 4c) [22], which provide better catalytic activity over the other porphyrin generations, according to Haber and co-workers [23].

Over the last few decades, a number of publications have covered the many catalytic activities of porphyrins; however, these studies were typically focused on methodology, converting only a few milli- or micrograms of substrates during the experiments. Herein, we intend to present porphyrin derivatives which are able to perform scaled-up transformations.

## 3. Oxidation Reactions with Porphyrin and Metalloporphyrin–Based Catalysts

Oxidation reactions are important synthetic tools, and a number of applications can be found in the chemical industry [24]. The manufacture of products obtained from oxidation of organic substrates and the production of pharmaceutical ingredients (APIs) are of major importance [25].

**Figure 5:**
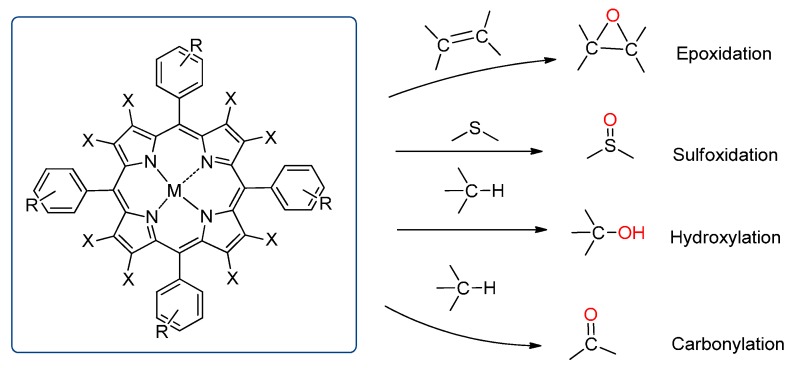
Some important transformations catalysed by porphyrins.

The inert nature of C–H bonds requires the use of highly reactive reagents and stoichiometric amounts of oxidizing agents such as strong inorganic acids, peroxyacids, or highly toxic oxo–metal oxidants; all yield products with low chemo, regio and stereoselectivity, and generate large amounts of toxic waste [26]. However, since the discovery of cytochrome P450, the use of metalloporphyrin catalysts has emerged as an alternative for controlled oxidation reactions in the above aspects (Figure 5) [27].

**Scheme 2:**
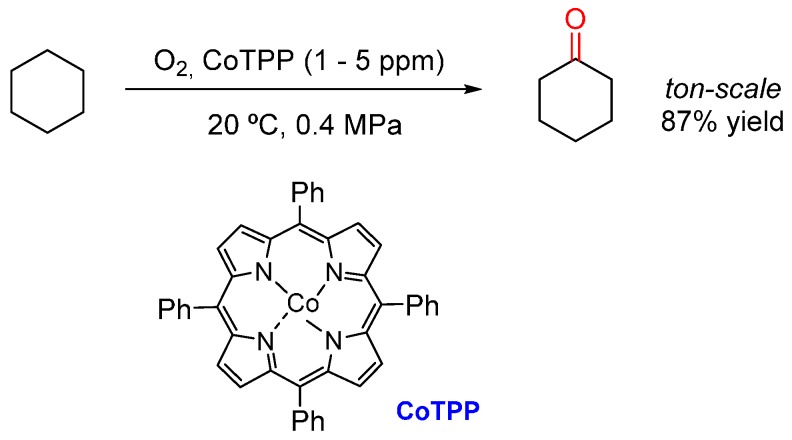
First industrial-scale bioinspired oxidation of cyclohexane with CoTPP.

This cytochrome family of enzymes play a key role in aerobic oxidation reactions in biological systems under mild conditions, such as highly selective hydroxylation of alkanes (C–O bond formation via saturated C–H bond functionalization) (Scheme 2) [28,29]. Metalloporphyrins with ruthenium, iron, manganese, among other metals, constitute the family of catalysts which are efficient to mediate C–H oxidations with high selectivity and good yields [30].

### 3.1. Epoxidation

**Scheme 3:**
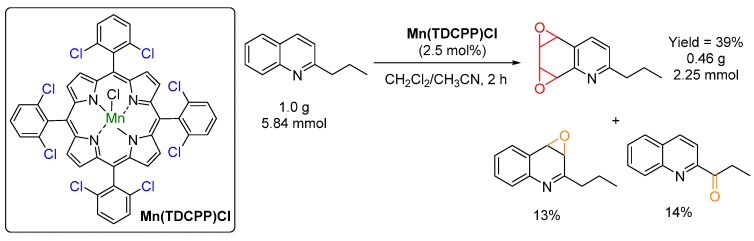
Metalloporphyrin catalysed bioinspired oxidation of 2-propylquinoline.

The epoxidation of alkenes is one of the processes of great importance in the fine chemical industry from an economic point of view, because epoxides are useful intermediates in the production and manufacture of high–value commercial polymers like polyurethane, polyamides, resins, and polyesters [31]. In addition, this transformation is used to carry out bioinspired oxidations [32] to produce drug candidates or metabolites (Scheme 3).

Iron porphyrins are effective catalysts for epoxidation of olefins by a number of oxidants, such as iodosylbenzene (PhIO) and 2,6-dichloropyridine-*N*-oxide (2,6-Cl_2_pyNO) [15]. Due to the relevance of cytochrome P-450 mechanism, catalytic epoxidation by iron porphyrins has gained great attention [33]. Since the Groves and co-workers’ first report in 1983 [34], several research groups have employed chiral metalloporphyrins [35] to catalyse the oxidation of various organic substrates, and new synthetic routes have been created to improve the catalytic performance of these heterocyclic complexes.

**Figure 6:**
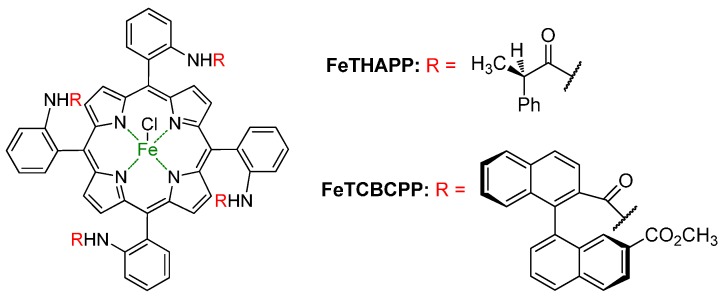
Chiral iron porphyrin catalysts for enantioselective epoxidation of olefins.

The complexes FeTHAPP and FeTCBCPP (Figure 6) are examples of metalloporphyrins which are able to perform asymmetric induction in the presence of either PhIO or iodosylmesitylene as oxygen donors, achieving products with enantioselectivities up to 50% and a turnover number (TON) near to 100 [36].

**Scheme 4:**
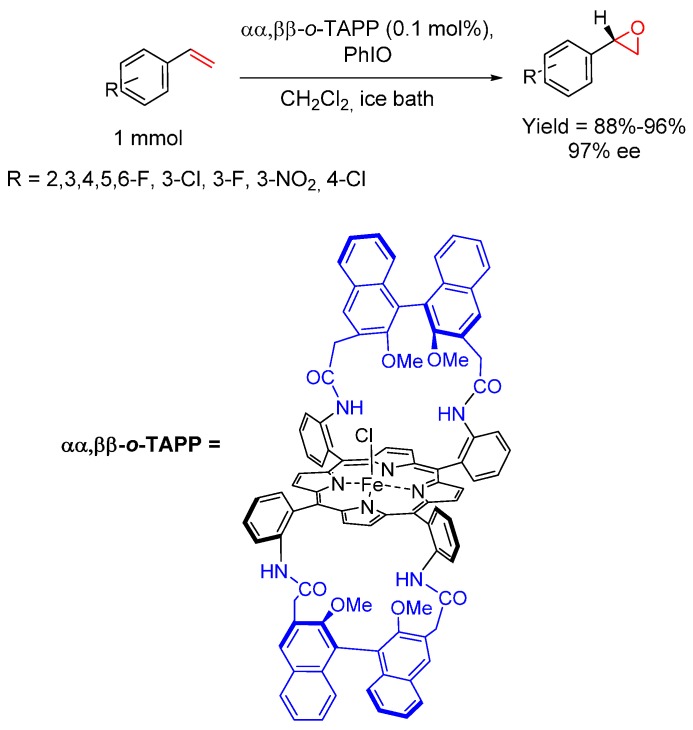
Enantioselective epoxidation of styrene derivatives.

Optically active epoxides obtained from the catalytic enantioselective epoxidation of alkenes are important intermediates in asymmetric synthesis, since these compounds are very useful for the synthesis of chirons with up to two contiguous stereogenic centres [37,38]. Chiral epoxidation of allylic alcohols provides interesting building blocks for the asymmetric synthesis of biologically active compounds, and consequently, asymmetric epoxidation of allylic alcohols has been extensively developed [39]. Chiral non-racemic iron porphyrins with binaphthyl moieties linked to the macrocycle allow the enantioselective epoxidation of non-functionalized terminal olefins with good enantioselectivity (>97% for styrene) and high turnover numbers (>16,000) (Scheme 4) [35,40].

In general, iron porphyrins present good reactivity to catalyse two primary reactions, namely the enantioselective transfer of an oxygen atom from the hydrogen peroxide to the substrate, and the decomposition of the peroxide in water and oxygen [41]. On the other hand, the use of manganese porphyrins as catalysts with hydrogen peroxide as oxidant, yield low enantiomeric excesses for epoxidations in organic solvents or biphasic medium [42].

**Scheme 5:**
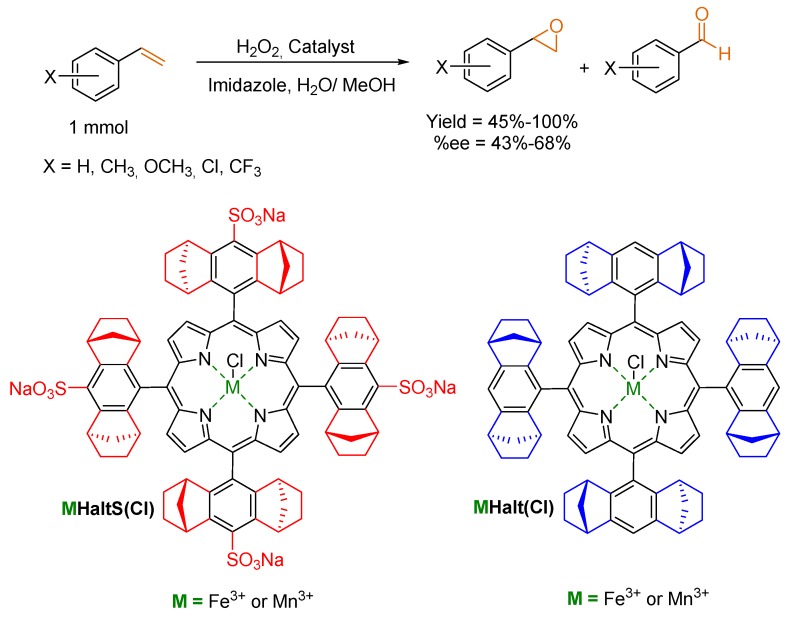
Epoxidation of styrenes using manganese porphyrins as catalyst and H_2_O_2_.

However, Simonneaux and co-workers reported in 2012 the enantioselective epoxidation of styrene derivatives using H_2_O_2_ (ee up to 68%), in water-methanol solutions using chiral water-soluble manganese and iron porphyrins as catalysts (Scheme 5) [43,44]. They also studied various factors which affect the catalytic epoxidation of olefins and found that the water present in the methanol is quite useful [44]. 

Besides the hydrogen peroxide decomposition [45] and other factors like the structural features of the porphyrin catalyst [46], the substrate concentration and the solvent [47] affect strongly the activity and the selectivity of these catalysts.

**Scheme 6:**
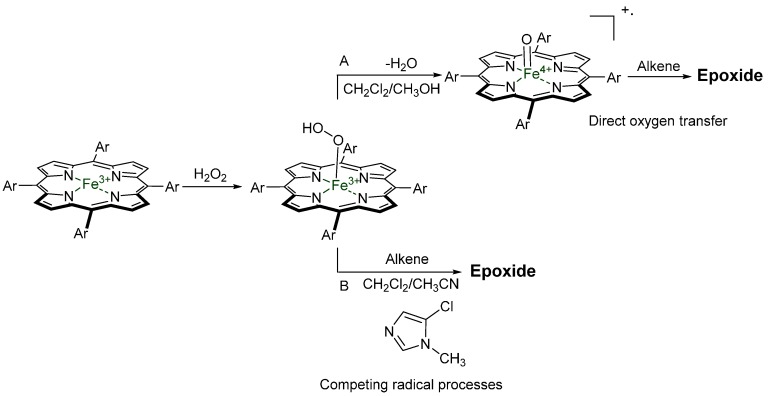
Different mechanisms in catalytic epoxidation depending on the conditions.

Scheme 6 shows how the reaction conditions affect the total conversion of alkenes when an iron porphyrin is employed as catalyst in a mixture of dichloromethane and methanol. In this case, direct oxygen transfer to obtain the epoxide is observed from the high-valent Fe(IV)-porphyrin radical cation complex (path A) [37]. On the other hand, when an aprotic solvent such as acetonitrile is used, the reactive intermediate is more likely to be the iron hydroperoxide complex, leading to the generation of competing radicals, thus promoting low selectivity and yield even though an imidazole is added to help the stabilization of the intermediate (path B) [37].

Despite numerous studies on the metalloporphyrin–catalysed epoxidation of olefins, the substrate types in such oxidation systems reported in the literature were rather limited and were usually confined to electron–rich alkenes such as styrenes, norbornene, cyclohexene and cyclooctene. However, employing dendritic ruthenium porphyrins [48] as catalysts, and 2,6-Cl_2_pyNO, *t*-BuOOH or O_2_, some authors have described efficient epoxidations of different alkenes in high yields and turnover numbers (>700).

**Scheme 7:**
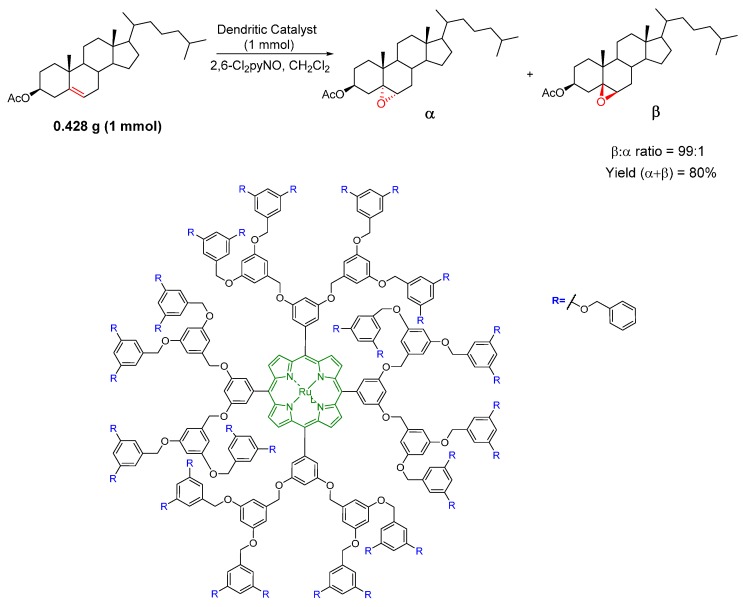
Dendritic Ru-porphyrin catalysed epoxidation of cholesteryl esters.

Che and co-workers investigated the catalytic properties of the catalyst for the epoxidation of unsaturated cholesteryl esters with 2,6-Cl_2_pyNO in dichloromethane using just 0.1 mol % of the dendritic ruthenium porphyrin catalyst (Scheme 7) [48].

### 3.2. Sulfides to Sulfoxides

**Scheme 8:**
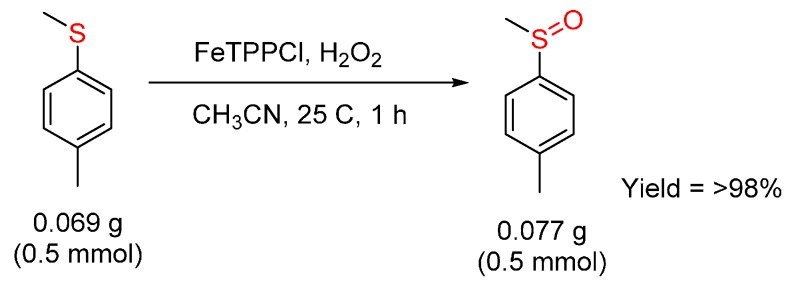
First example of a bioinspired sulfoxidation.

Since the pioneering work of Oae and co-workers (Scheme 8) [49] on the bioinspired oxidation of sulfides to sulfoxides, this transformation has gained much attention. Historically, sulfur-containing compounds have figured as targets for their importance to the pharmaceutical industry as antibacterial agents [25]. Therefore, the development of catalytic systems for the preparation of optically active sulfoxides is important, because they are chiral synthons [50] in the synthesis of bioactive compounds [51].

**Scheme 9:**
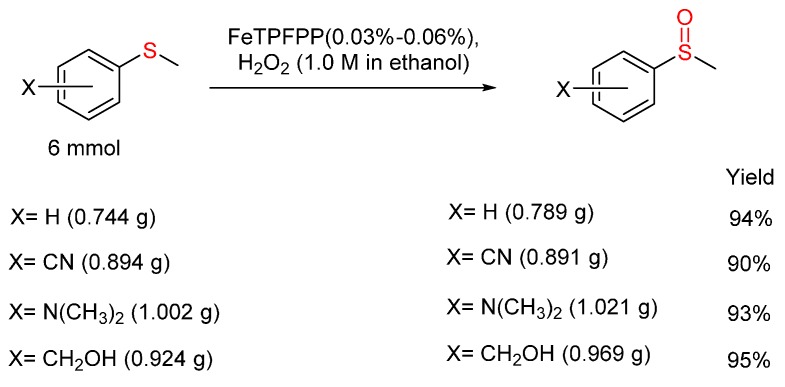
Synthesis of sulfoxides from sulfides.

Sulfoxidation is commonly presented as being a direct pathway for generating sulfoxides, however, most of the reagents used for this reaction such as iodosylbenzene, peroxyacids, and stoichiometric oxo-metal oxidants are unsatisfactory due to their high toxicity and low chemoselectivity between sulfoxide and sulfone products [52]. One successful example of a green protocol was described by Baciocchi and co-workers, who reported the oxidation of sulfides with an ethanolic solution of H_2_O_2_ and iron tetrakis(pentafluorophenyl)porphyrin as catalyst, thus giving the corresponding sulfoxides on a gram-scale (Scheme 9) [53].

**Scheme 10:**
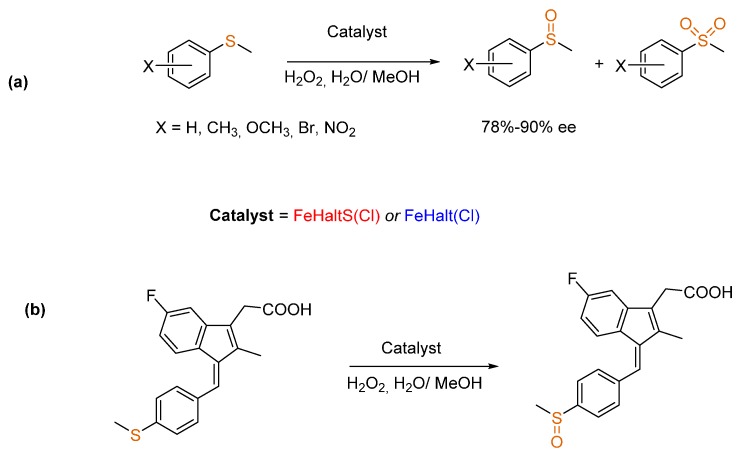
(**a**) Enantioselective sulfoxidation of styrenes using iron porphyrins as catalyst and hydrogen peroxide; (**b**) Metalloporphyrin-catalysed epoxidation for the enantioselective synthesis of Sulindac^®^.

Among the methods to obtain sulfoxides [54], the enantioselective oxidation of sulfides with small amounts of metal-organic catalysts is one of the most attractive routes to optically active sulfoxides [55]. For example, Simonneaux and co-workers reported a small scale enantioselective sulfide oxidation with 35% aqueous hydrogen peroxide and a chiral water-soluble iron-porphyrin as catalyst [56], thus obtaining sulfoxides with enantioselectivities between 78% and 90% from alkyl aryl sulfides, bearing electron-withdrawing groups in the phenyl ring (Scheme 10a).

The advantages of the process is supported by the absence of excess of oxidant, small reaction time, reaction at room temperature, and protection against destruction of the macrocycle by the two bulky norbornane groups joined to the central benzene ring [56]; however, this procedure was not scaled-up, and only a few micrograms of sulfide were converted.

Another example of an enantioselective oxidation of sulfides (small scale) catalysed by a chiral manganese porphyrin in an aqueous methanol solution in the presence of H_2_O_2_ furnished the non-steroidal anti-inflammatory drug Sulindac^®^ (Banyu Pharmaceutical Co., Ltd., Tokyo, Japan) (Scheme 10b) [57].

**Scheme 11:**
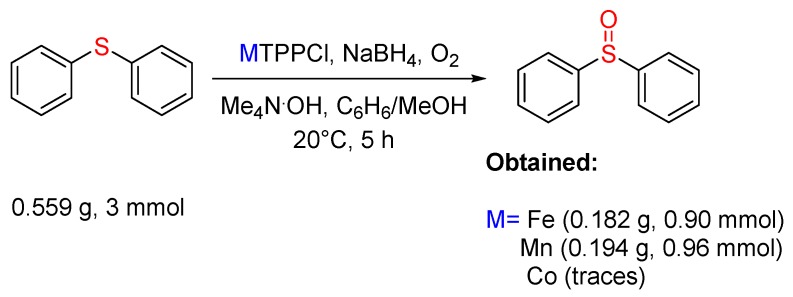
Synthesis of sulfoxides from sulfides using the MTPPCl/NaBH_4_/Me_4_N^.^OH system.

Nonetheless, the formation of sulfoxides using metalloporphyrins as catalyst can be achieved by other methodologies, but in this case, on a superior scale. The use of oxygen as the oxidant with NaBH_4_ and Me_4_NOH, [58] is an example to obtain the oxidized product of diarylsulfide compounds (Scheme 11).

### 3.3. Hydroxylation

Once the mechanism by which the cytochrome P-450 acts as catalyst in C–H functionalization became known [59], with an iron porphyrin core as the active site, many synthetic metalloporphyrins have been studied as catalyst for this purpose with different organic substrates [60,61]. In fact, this is one of the most difficult transformations for the petrochemical industry, and one of the major challenge to convert petroleum derivatives into chemicals of higher value [62].

**Scheme 12:**
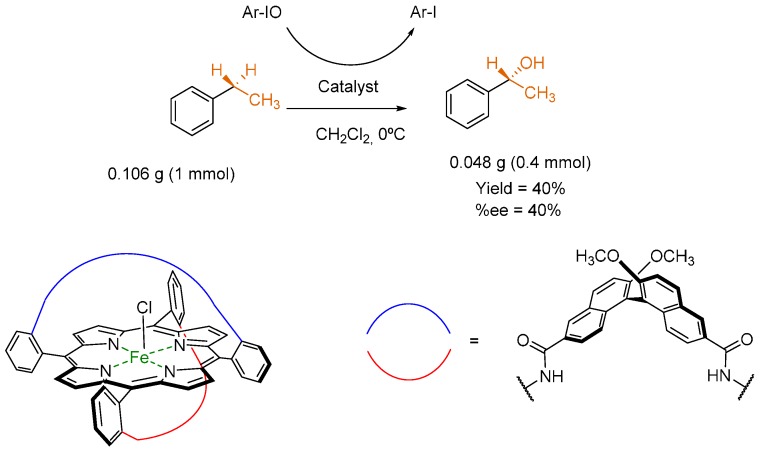
Enantioselective hydroxylation of ethylbenzene by a chiral iron-porphyrin.

The first report of metalloporphyrin-catalysed hydroxylation of saturated C–H bonds was published by Groves and co-workers in 1979 [17], where they showed the catalytic activity of [Fe(TPP)Cl] towards oxidation of cyclohexane and adamantane with iodosylbenzene to give the corresponding alcohols. Approximately 10 years later, Groves and Viski reported for the first time, the enantioselective hydroxylation of ethylbenzenes catalysed by a chiral iron porphyrin with up to 77% ee using PhIO as oxidant (Scheme 12) [63,64]. When manganese was used, higher yields were obtained, but the enantioselectivity decreased and ketones were also observed as by-products in the catalytic reactions.

**Scheme 13:**
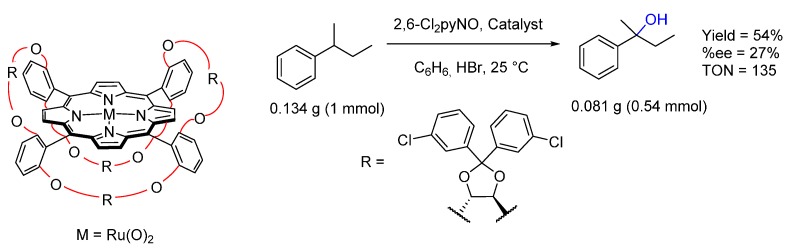
Catalytic enantioselective hydroxylation of a tertiary alkane.

In 1999, Gross and Ini reported the first example of ruthenium-catalysed enantioselective (ee up to 38%) hydroxylation of racemic tertiary alkanes, using a chiral porphyrin ligand and 2,6-Cl_2_pyNO as oxidant (Scheme 13) [65].

**Scheme 14:**
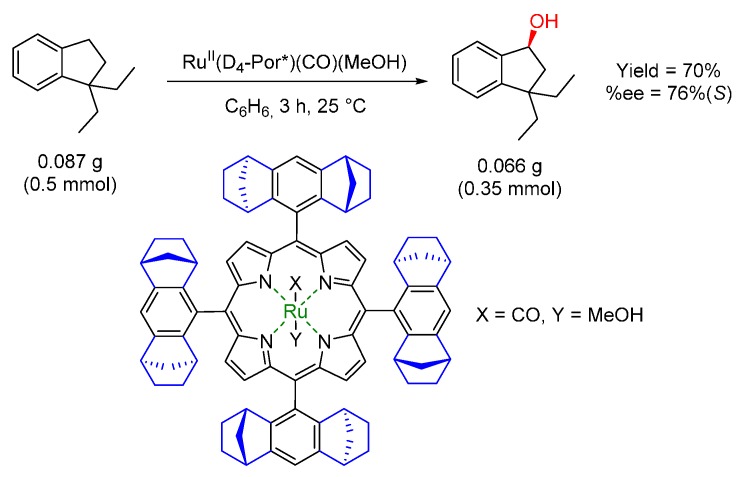
Ruthenium catalysed asymmetric alkane hydroxylation.

Che and co-workers [66] reported the Ru-catalysed enantioselective hydroxylation of aromatic hydrocarbons with benzylic C–H bonds. Using 2,6-Cl_2_pyNO as oxidant, the chiral ruthenium porphyrin was shown to be an effective catalyst for hydroxylating a series of aromatic hydrocarbons to form the corresponding secondary alcohols. Although the conversion of the substrates was incomplete, the Ru-based hydroxylation gave yields up to 76% ee (Scheme 14).

**Scheme 15:**
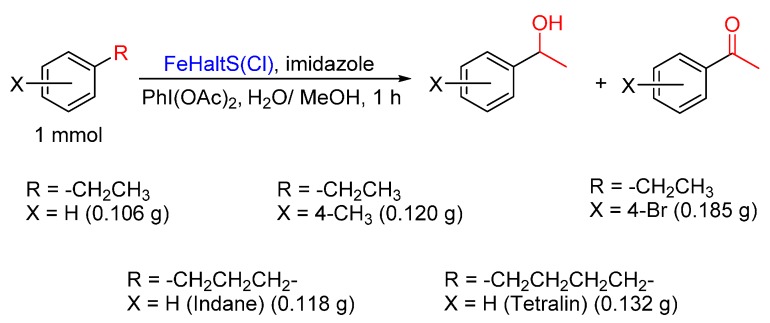
Hydroxylation of ethylbenzene derivatives using a water-soluble iron-porphyrin as catalyst.

In 2012 Simonneaux and co-workers reported examples of enantioselective hydroxylation of alkanes, with a chiral iron porphyrin as catalyst and using hydrogen peroxide as oxidant in methanol and water, to give optically active secondary alcohols (ee up to 63%) [43]. Nonetheless, one limitation for this system is the requirement of an excess of alkane versus the oxidant, and consequently the asymmetric hydroxylation of alkanes using these conditions is still difficult to proceed without electron-deficient chiral metalloporphyrins [44]. The treatment of ethylbenzene with H_2_O_2_ and catalysis by the complex (Scheme 15) afforded a mixture of 1–phenylethanol (57%) and acetophenone (43%) with 88% of conversion [43].

**Scheme 16:**
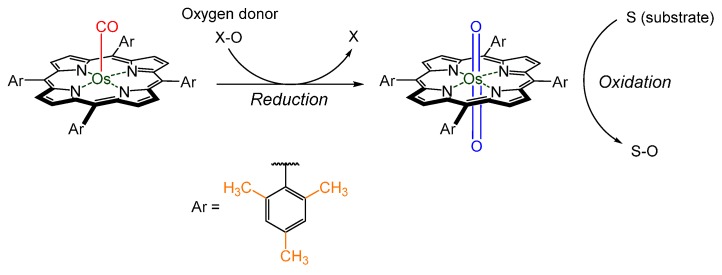
Representation of the oxidation process by the osmium-porphyrin [70].

Other types of porphyrins have been explored for this hydroxylation reaction, and in 2007, Idemori and co–workers studied Mn(III)–tetrapyridylporphyrin as catalyst in the hydroxylation of cyclohexene [67]. Iida and co-workers have shown that the combination of *tert*-butyl hydroperoxide (TBHP) and the osmium(II) carbonyl complex of *meso*-tetramesitylporphyrin [Os(TMP)(CO)] as oxygen donor and catalyst respectively (Scheme 16), is an efficient and versatile oxidation system for the functionalization of bioactive molecules, like bile acids [68] and terpenoids [69].

**Scheme 17:**
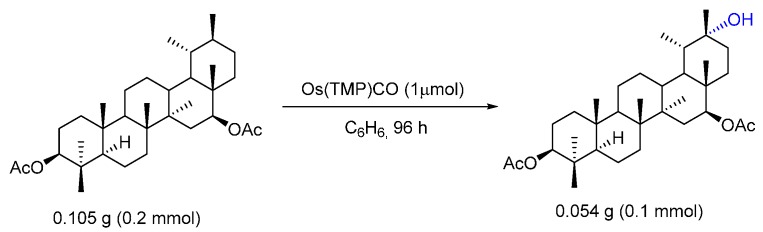
Hydroxylation of dihydrofaradiol diacetate with the osmium porphyrin.

The oxy-functionalization system with osmium-porphyrins as catalyst regioselectively hydroxylates one of the inactivated tertiary C–H bonds in dihydrofaradiol diacetate to form the corresponding alcohol in 50% yield (Scheme 17). The functionalization of triterpenoids with the oxidant system afforded a variety of novel oxygenated derivatives in one-step with good isolated yields [69].

### 3.4. Oxidation of Alcohols to Carbonyl Compounds

**Scheme 18:**
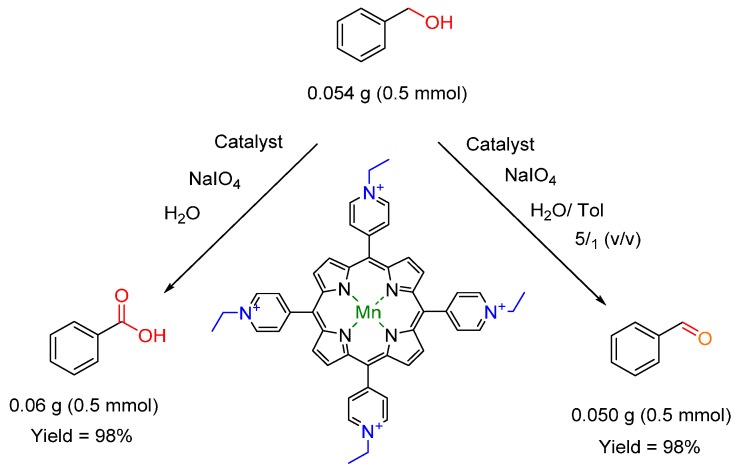
Oxidation of benzylic alcohols to carbonyl compounds with a Mn-porphyrin.

The transformation of primary alcohols to the corresponding aldehydes or carboxylic acids is important for organic synthesis, since these are versatile functional groups which are present in many building blocks [71] (Scheme 18).

Conventionally, stoichiometric or even over-stoichiometric amounts of metal oxides and metal complexes are used for these oxidations [72], but the catalytic oxidation with O_2_ is of sustainability interest [73]. 

Metalloporphyrins have been widely used as catalysts for various oxidations of alcohols with PhIO [74], 2,6-Cl_2_PyNO [75], *m*-chloroperbenzoic acid [76], Bu_4_NHSO_5_ (tetrabutylammonium peroxymonosulfate) [77] and oxygen as oxidants. Woo and co-workers reported the aerobic homogeneous oxidation of benzyl alcohol with oxo-titanium porphyrin ((TPP)Ti=O), which gave benzaldehyde in 50% yield [78]. It has been found that (TPP)Ti=O reacts with free diols to yield diolato complexes [79], and these complexes undergo oxidative cleavage reactions at high temperatures to release carbonyl compounds.

**Scheme 19:**
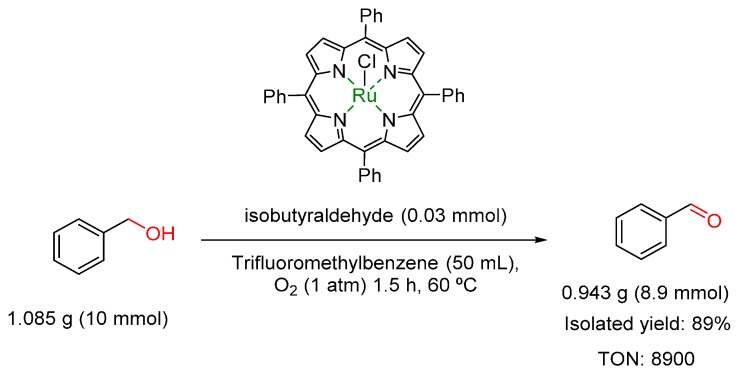
Gram-scale oxidation of benzylic alcohols with Ru(TPP)Cl.

Ji and co-workers reported a selective oxidation of alcohols to carbonyl compounds by molecular oxygen with isobutyraldehyde as oxygen acceptor in the presence of ruthenium (III) *meso*-tetraphenylporphyrin chloride (Ru(TPP)Cl) (Scheme 19) [80]. In addition, they found that the catalytic activity and selectivity of metalloporphyrins towards benzaldehydes appeared to be dependent on the nature of the central ion, and were influenced by the stability of different valences of the metal atoms and their respective electric potential. 

Oxidation of aldehydes to the corresponding carboxylic acids is one of the ubiquitous transformation in organic synthesis, for the preparation of numerous APIs, vitamins and fragrances [73]. Rebelo and co-authors investigated the oxidation of benzaldehyde with hydrogen peroxide using a Mn(III) porphyrin/ammonium acetate system to give benzoic acid in 93% yield [81].

**Scheme 20:**
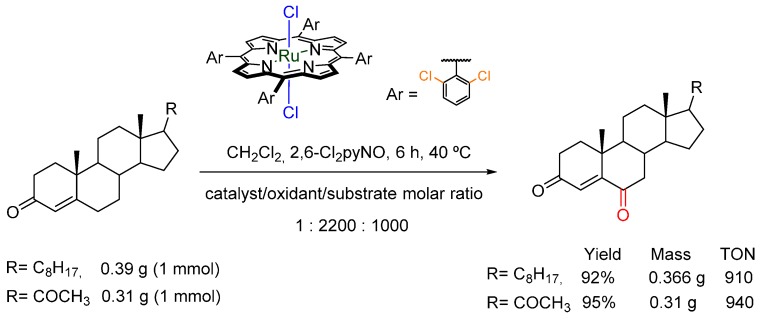
Oxidation of ketosteroids catalysed by ruthenium 2,6-TDCPP dichloride.

The oxidation of α,β-enones at the γ position is a relevant transformation, with few known methodologies. Che and co-workers reported the Ru-porphyrin catalysed oxidation of ketosteroids to form diketosteroids using *meso*-tetrakis(2,6-dichlorophenyl)porphyrin (Ru(TDClPP)Cl_2_) as catalyst and 2,6–Cl_2_pyNO as oxidant (Scheme 20) [82].

## 4. Porphyrin Derivatives Acting as Photocatalysts

**Figure 7:**
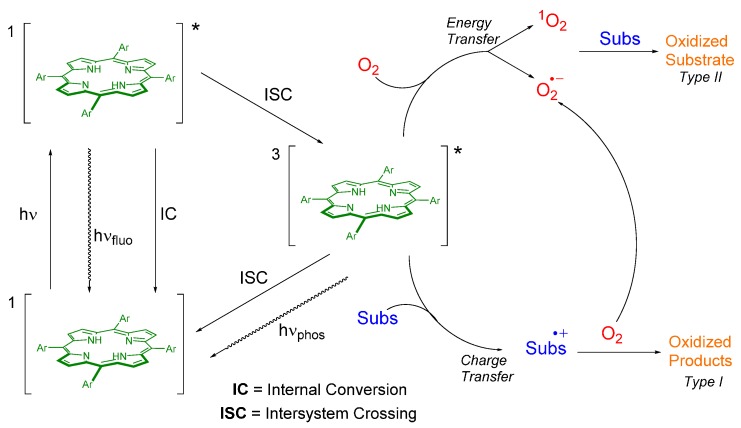
Representation of the mechanism of porphyrin photosensitized reactions.

Since 1930, when Kautsky first examined the generation of singlet oxygen {O_2_ (^1^Δ_g_)}, by energy transfer from a photoexcited organic molecules to molecular oxygen, this kind of environmentally friendly methodology has been of interest [83]. The importance of singlet oxygen in many physical and biological processes has been recognized, with rich details concerning the physics and chemistry of this electronically excited molecule [84]. Generally, singlet oxygen can be generated by chemical or photosensitized reactions, but the photochemical approach is more cost-competitive. The mechanism of the photochemical singlet oxygen generation includes the promotion of the photosensitizer to the triplet state, passing through a singlet -excited state. At the triplet state many photosensitizers are able to transfer energy to molecular oxygen leading to the formation of reactive oxygen species (ROS) (Figure 7) [85].

In this context, porphyrins are prominent photosensitizers because of their high light absorption coefficient, exited state energy levels, and high photostability as compared to other dyes. For example, *meso*-tetraphenylporphyrin (TPP) has been chosen as one of the most highly effective photosensitizer [86] for singlet oxygen generation [87]. 

**Scheme 21:**
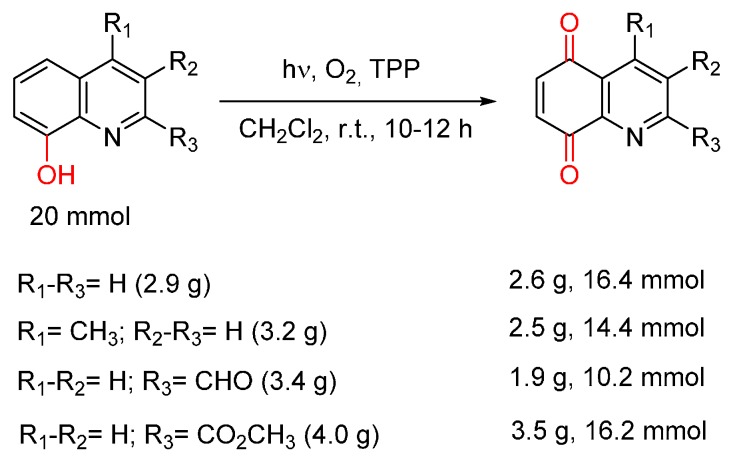
Synthesis of substituted quinoline-5,8-quinones by photooxygenation.

In 1999, methylene blue was used as photosensitizer in the photooxidation of 8-hydroxyquinoline to afford quinoline-5,8-quinone in 64%–70% yields [88]. However, Cossy and Belotti showed in 2001 an improved method using TPP for the photooxygenation of substituted 8-hydroxyquinolines in 50%–89% yield (Scheme 21) [85]. 

**Scheme 22:**
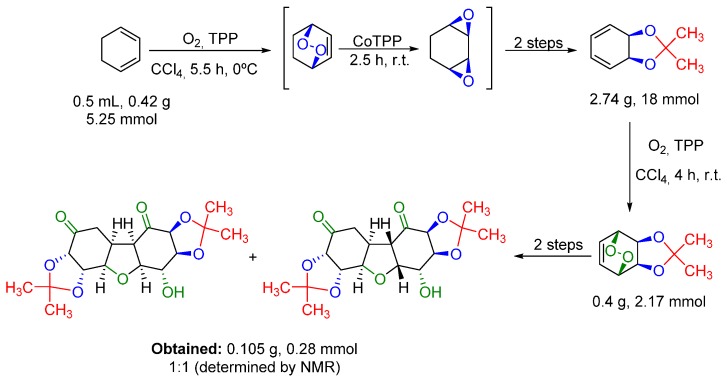
Synthesis of decahydrodibenzofurans by using porphyrins as catalysts.

Spivey and co-workers reported in 2010 the synthesis of high-functionalized molecules using two photooxygenation reactions with oxygen, visible light and TPP as photocatalyst, including a base-catalysed Kornblum DeLaMare rearrangement. Also, Co(II)TPP was used three times as the oxidation catalyst (Scheme 22) showing its versatility in the entire synthetic approach [89].

**Scheme 23:**
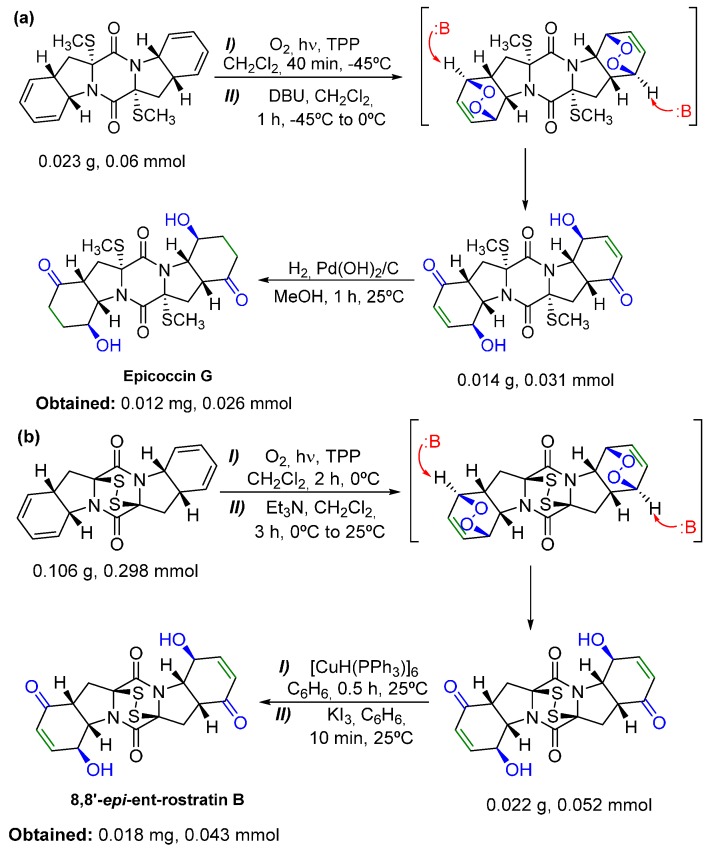
Synthesis of bisendoperoxide intermediates in the total synthesis of (**a**) epicoccin G and (**b**) 8,8’-*epi*-ent-rostratin B using TPP as photosensitizer.

**Scheme 24:**
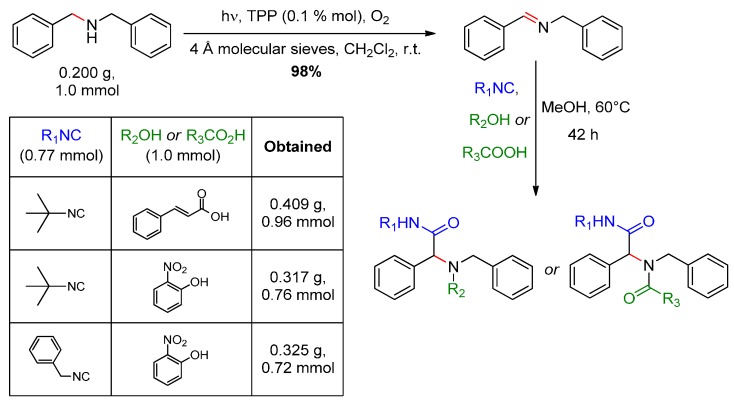
Photooxidation of secondary benzylamines to imines.

By comparison, in 2011 Nicolaou presented the total synthesis of dithiodiketopiperazines (epicoccin G and 8,8’-*epi-ent-*rostratin B) including two TPP-photocatalysed endoperoxide formations, and base-catalysed Kornblum DeLaMare rearrangements (Scheme 23) [90]. Singlet oxygen generation by porphyrins can take place in reactions with amines [91]. In the case of secondary amines, there is an example with the dehydrogenation of *N*–neopentylallylic amine using TPP as photocatalyst to obtain the corresponding imine product [92]. Moreover, Che and co-workers [93] reported the highly efficient photooxidation of secondary benzylamines to imines in 90% yield using molecular oxygen and TPP as photosensitizer. They used directly the *in situ* formed imine products for further functionalization to develop an oxidative Ugi–type MCR (Scheme 24).

**Scheme 25:**
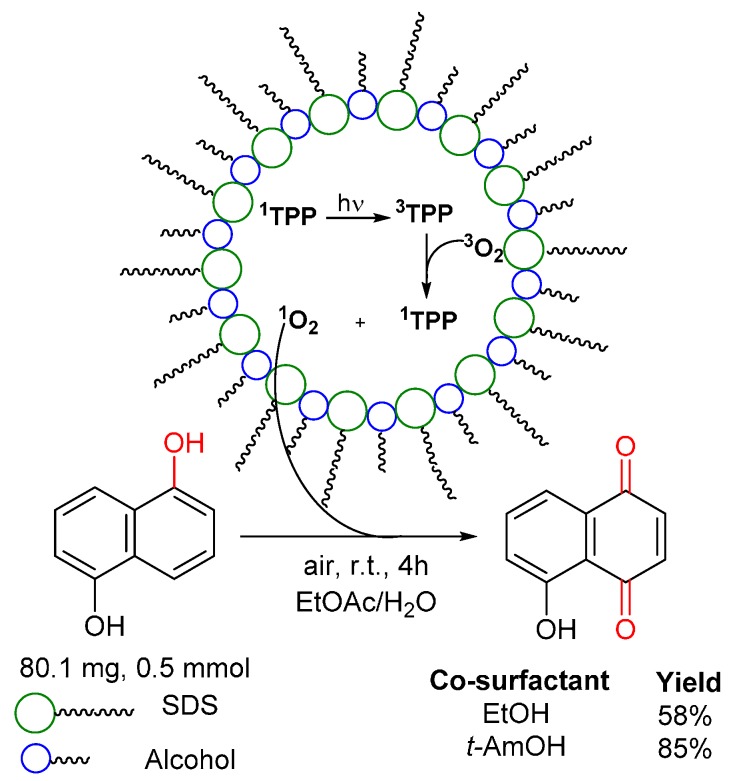
Schematic representation of a “green” microemulsion Juglone synthesis.

Another photocatalytic application of porphyrins is their encapsulation in microemulsions of ethyl acetate, water and surfactants to allow the use of TPP as photosensitizer for singlet oxygen generation [94]. For example, Oelgemöller and co-workers carried out the photooxygenation of 1,5-dihydroxynaphthalene to Juglone (5-hydroxy-1,4-naphthoquinone) as a reaction model in a micellar system, but only on a small-scale (Scheme 25) [95].

### 4.1. Immobilized Porphyrins as Photocatalysts

One approach to enhance the usefulness of metalloporphyrin catalysis, involves recyclability through heterogeneous catalytic systems [96] by using immobilized homogeneous catalysts [97]. In the case of asymmetric oxidation, immobilization can avoid the need for chiral ligand recovery. One of the simplest ways to prepare a polymer-immobilized catalyst is the direct reaction of a simple functionalized polymer, such as Merrifield`s resin with a derivative of the desired ligand and then insertion of the metal [98].

**Scheme 26:**
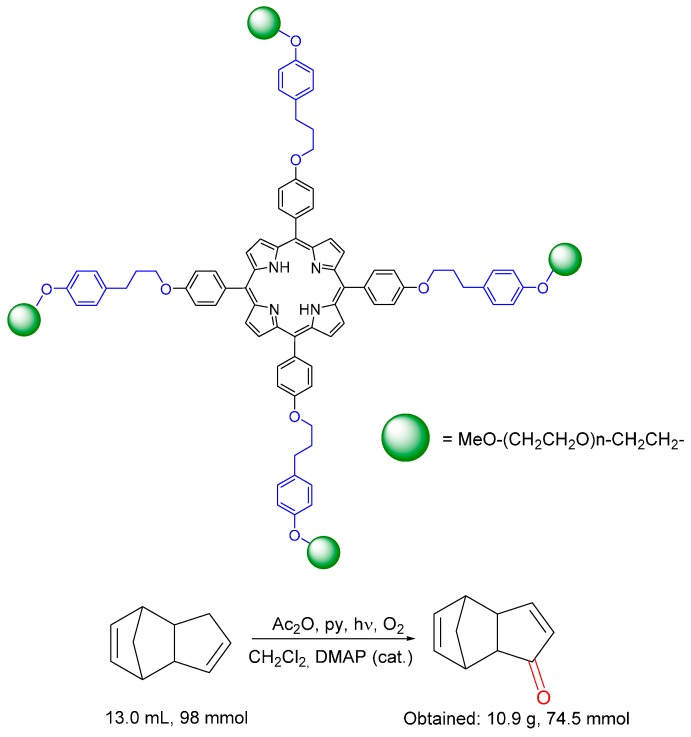
PEG-supported tetrahydroxyphenyl porphyrin for photooxidation reactions.

The covalent immobilization of porphyrins on polymeric matrices is very versatile to generate singlet oxygen [99]. Anchoring the photocatalyst onto a poly(ethylene glycol) allows the easy recovery of the catalyst from the reaction mixture, as Benaglia and co-workers [100] reported with a new poly(ethylene glycol)-supported porphyrin which exhibited high activity as catalyst, comparable to that of a non-anchored sensitizer (Scheme 26).

**Scheme 27:**
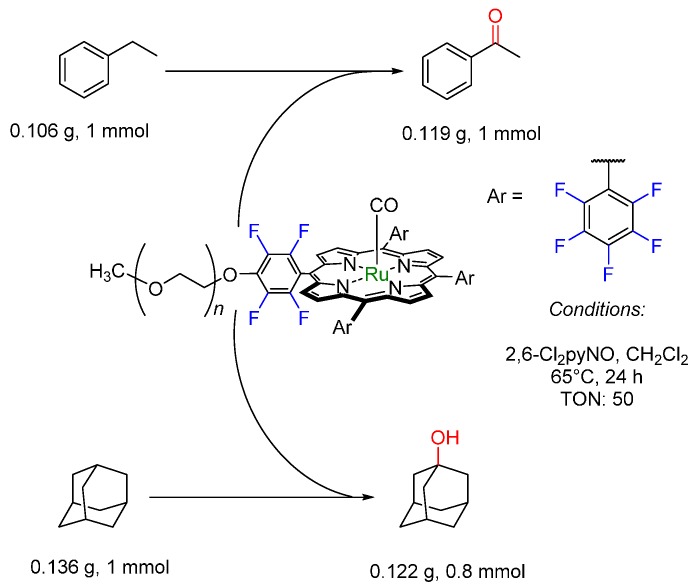
PEG-supported ruthenium porphyrin for oxidation reactions.

Che and co-workers reported a covalently attached carbonylruthenium(II)*meso*-tetrakis(pentafluorophenyl)porphyrin (Ru(TPFPP)(CO)) linked to hydrophilic PEG macromolecules [101]. The resulting PEG-supported Ru-porphyrin was shown to be an effective catalyst for oxidation of both secondary and tertiary C–H bonds (Scheme 27) [101].

**Scheme 28:**
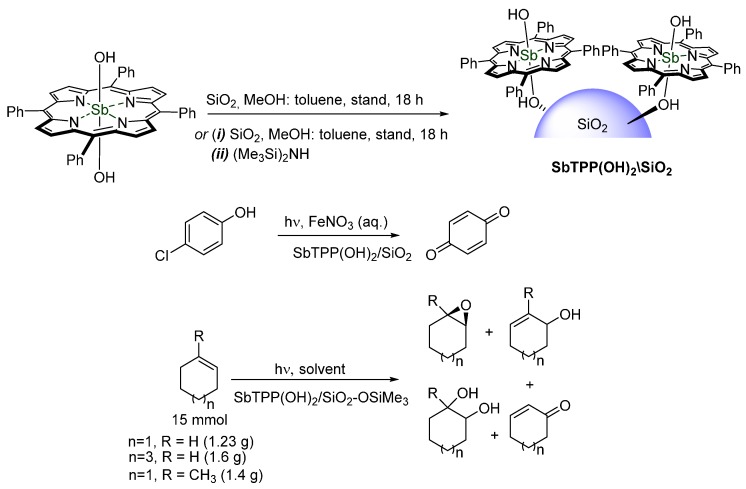
Different photocatalytic properties of antimony SbTPP(OH)_2_\SiO_2_ supported over silica.

In 2004, Shiragami and co-workers reported a scaled-up photocatalytic oxidation of cycloalkenes with O_2_ under visible-light using a SbTPP(OH)_2_\SiO_2_ catalyst (Scheme 28) [102]. 

**Scheme 29:**
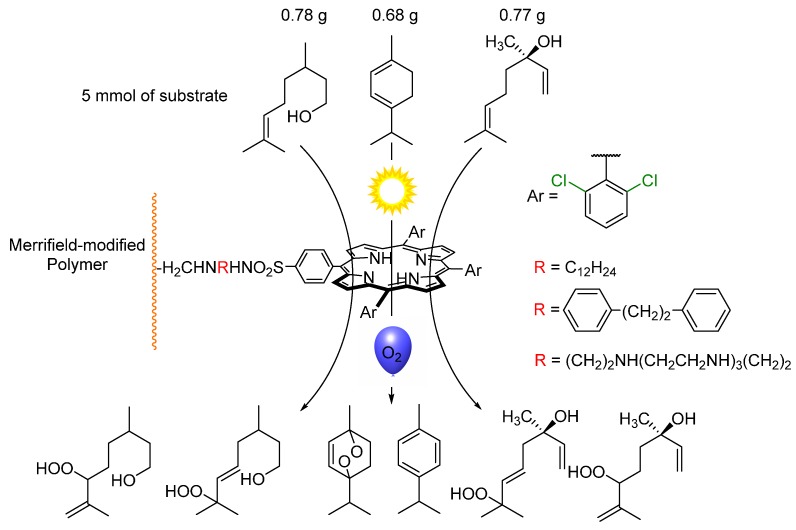
Solar photooxygenation with supported porphyrins on Merrifield-modified polymers.

Recently, Gonsalves and co-workers have synthesized supported materials with a non-symmetric halogenated tetra-arylporphyrin linked to Merrifield polymers, thus showing high efficiency in the scalable photooxygenations of different substrates using sunlight (Scheme 29) [103,104]. 

### 4.2. Continuous Flow Photocatalysis

Continuous flow reactors have recently emerged as a new technology in chemical synthesis with a wide variety of efficient applications [105,106]. The small inner dimensions of these devices in combination with their continuous flow operation make them especially attractive for photochemical reactions [107]. A combination of a microreactor system as the reaction medium and visible light offers a new and convenient approach towards green photochemistry, in which the production of by-products is also reduced. As a result, not only enhanced selectivity is achieved, but also the amount of environmentally problematic and expensive solvents are reduced, and the security is really improved [108].

Reactions with singlet oxygen (^1^O_2_) have not been used for large/industrial scale processes for the following reasons: (I) decreased photochemical efficiency of photosensitizers in batch conditions due to low light penetration in classic photochemical reactors); (II) the need for high substrate dilutions due to the safety requested by procedures with peroxides and endoperoxides formation; (III) major by-products formation; and (IV) difficulty to find suitable non-flammable and environmentally friendly solvents, with no incompatibility with singlet oxygen generation [109]. 

**Scheme 30:**
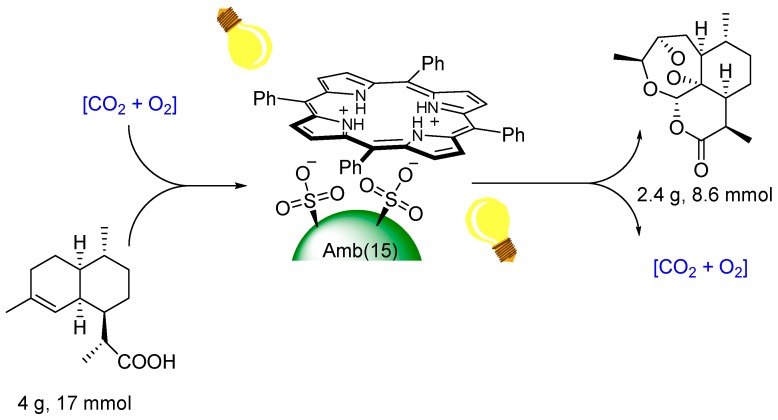
One-pot semi-synthesis of artemisinin using immobilized TPP on Amberlyst-15.

However, the use of supercritical carbon dioxide as solvent brings a solution to some of these issues, because it is neither flammable nor toxic, completely miscible with gases, and has a lower viscosity and higher diffusivity [110]. For example, George and co-workers reported the production of artemisinin (gram-scale) using liquid CO_2_ as solvent and an immobilized porphyrin as photocatalyst. They developed a simple method to anchor TPP or TPFPP onto the sulfonated cross-linked polystyrene ion-exchange resin (Amberlyst-15) [111]. This yielded a dual catalyst, with both the Bronsted acid and photo-catalytic functions needed to convert dihydroartemisinic acid (DHAA) into artemisinin, currently a very important drug for malaria treatment (Scheme 30). The reaction resulted in 98% of conversion using toluene as co-solvent.

**Scheme 31:**
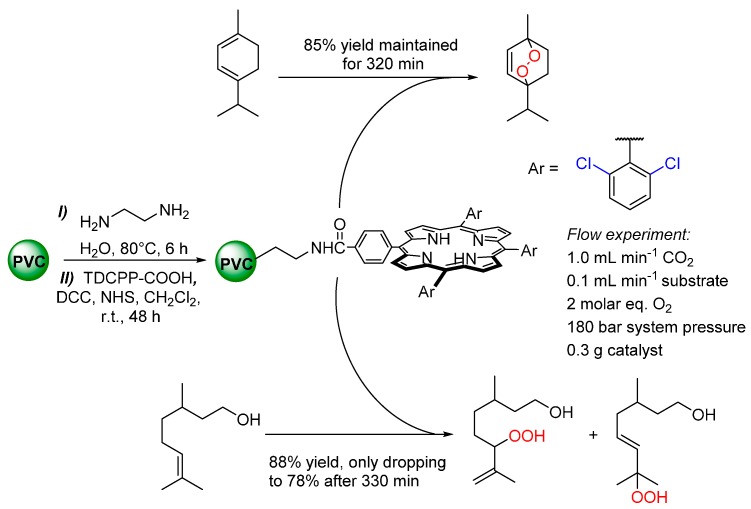
Continuous flow reactions with PVC amino-functionalized covalently bonded porphyrin.

George and co-workers have been the pioneers in using this kind of green solvent approach, since in 2011 they reported the use of supercritical carbon dioxide as solvent with different immobilized photosensitizers for the continuous flow synthesis of ascaridole from α-terpinene (Scheme 31) [112]. The authors coupled a porphyrin to amino functionalized PVC beads by covalent amide linkage, and achieved the continuous production of ascaridole and also citronellol hydroperoxide from citronellol [112].

**Scheme 32:**
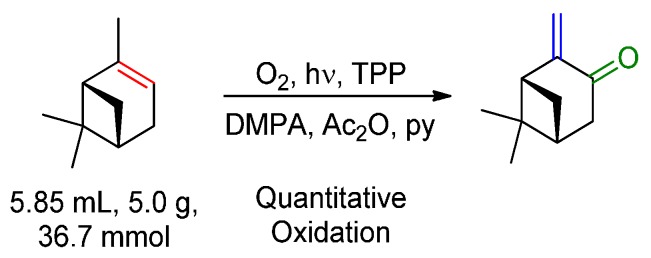
Scalable photochemical conversion of α-pinene to pinocarvone.

Lapkin and co-workers[113] have also carried out a gram-scale photooxygenation/rearrangement of α-pinene using TPP as sensitizer in dichloromethane under continuous flow conditions, thus obtaining pinocarvone quantitatively. This oxygenation reaction was efficiently performed in a segmented gas-liquid flow condition in a very safe manner (Scheme 32) [113].

**Scheme 33:**
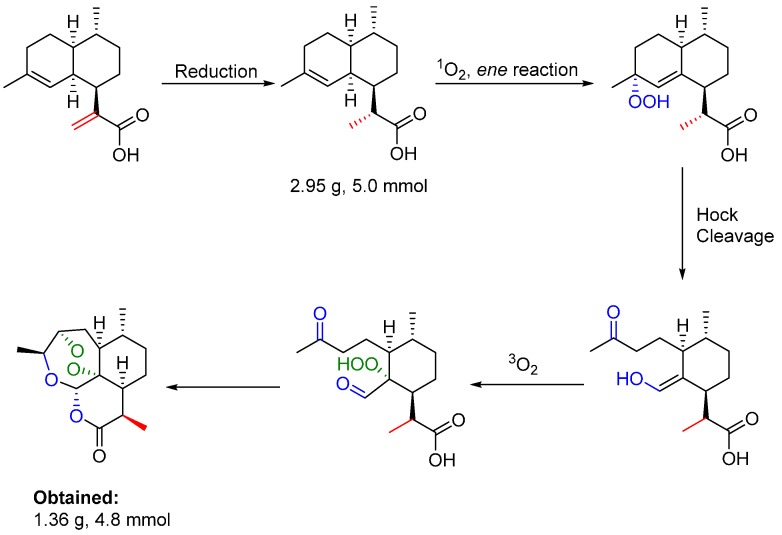
Synthetic route to artemisinin from artemisinic acid.

Seeberger and co-workers have also established gram-scale photoinduced singlet-oxygen generation under continuous flow by using TPP as photocatalyst, and a very simple photoreactor (perfluoroalkoxy—PFA—tube coiled around a cooled glass tube and a lamp) [114]. In this apparatus, the illumination of the entire solution is highly efficient because the tubing has a constant, narrow diameter, and it is positioned very close to the light source. Thus, an increasing quantity (space-time yield = 200g·day^-1^) of the product can be produced in long-term experiments (Scheme 33). 

The above mentioned method has opened perspectives to carry out the crucial step in the synthesis of artemisinin (Scheme 33) [115]. The great importance of TPP as photosensitizer for singlet oxygen production can be highlighted by the green gram-scale synthesis of artemisinin recently established as an industrial process by the pharmaceutical company Sanofi [116].

**Scheme 34:**
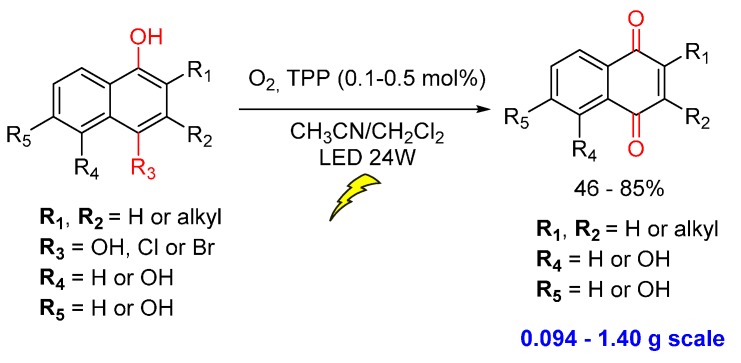
Photo-oxidation of naphthols under continuous flow conditions.

To finish this section, it is important to highlight the recent and cost competitive protocol for photooxygenation of naphthols under continuous flow conditions, which was recently published by Oliveira, Miller and McQuade [117]. A careful methodological study was performed using four different porphyrins as photocatalysts. After optimisation of the reaction conditions, a number of substituted naphthols were converted into the corresponding naphthoquinones in high yields, as well as in gram-scale experiments (up to 1.4 g produced continuously in a 24 h experiment) (Scheme 34).

## 5. Perspectives

Over the last few decades, synthetic organic chemists have taken advantage of the catalytic activity of porphyrin derivatives to develop novel laboratory procedures and to enhance existing protocols, but normally on a small scale. Recently some scaled-up reactions have been described showing that these macrocycles are very useful for increasing the scope of available multiple catalysts. The challenge now is to explore the broad potential and applications of these catalysts for the synthesis of important products from an economic point of view, for the industrial and pharmaceutical industries. 

In addition, another challenge is to introduce simple and cost-competitive synthetic routes [118] to access porphyrinods, since historically these compounds are considered rare due to low synthetic yields and tedious purification protocols. Therefore, it is necessary to incorporate enabling techniques for the optimisation of the production and utilization of these fascinating naturally inspired catalysts, and chemists must now consider these robust compounds for many new purposes.

## References

[1] Wisniak J. (2010). The history of catalysis. From the beginning to Nobel prizes. Educ. Quim..

[2] Roberts M.W. (2000). Birth of the catalytic concept (1800–1900). Catal. Lett..

[3] Van Santen R., Beller M., Renken A., van Santen R., Beller M., Renken A., Van Santen R. (2012). Catalysis in perspective: Historic review. Catalysis: From Principles to Applications.

[4] Drain C.M., Hupp J.T., Suslick K.S., Wasielewski M.R., Chen X. (2002). A perspective on four new porphyrin-based functional materials and devices. J. Porphyrins Phthalocyanines.

[5] Chalmers M., Notman N. (2009). Living the nobel life. Chem. World.

[6] Zhou Q.L. (2015). Transition-metal catalysis and organocatalysis: Where can progress be expected?. Angew. Chem..

[7] Carvalho C.M.B., Brocksom T.J., de Oliveira K.T. (2013). Tetrabenzoporphyrins: Synthetic developments and applications. Chem. Soc. Rev..

[8] Betoni Momo P., Pavani C., Baptista M.S., Brocksom T.J., de Oliveira K.T. (2014). Chemical transformations and photophysical properties of *meso*-tetrathienyl-substituted porphyrin derivatives. Eur. J. Org. Chem..

[9] Carvalho C.M.B., Fujita M.A., Brocksom T.J., de Oliveira K.T. (2013). Synthesis and photophysical evaluations of β-fused uracil-porphyrin derivatives. Tetrahedron.

[10] Perutz M.F. (1980). Stereochemical mechanism of oxygen transport by haemoglobin. Proc. R. Soc. Lond. B. Biol..

[11] Senge M.O., MacGowan S.A., O’Brien J.M. (2015). Conformational control of cofactors in nature—The influence of protein-induced macrocycle distortion on the biological function of tetrapyrroles. Chem. Commun..

[12] Milgrom L.R. (1997). The Colours of Life—An Introduction to the Chemistry of Porphyrins and Related Compounds.

[13] Battersby A.R. (2000). Tetrapyrroles: The pigments of life. Nat. Prod. Rep..

[14] Sheftel A.D., Mason A.B., Ponka P. (2012). The long history of iron in the Universe and in health and disease. Biochim. Biophys. Acta—Gen. Subj..

[15] Sheldon R., Sheldon R. (1994). Metalloporphyrins in Catalytic Oxidations.

[16] Collman J., Zhang X., Lee V., Uffelman E., Brauman J. (1993). Regioselective and enantioselective epoxidation catalysed by metalloporphyrins. Science.

[17] Groves J.T., Nemo T.E., Myers R.S. (1979). Hydroxylation and epoxidation catalysed by iron-porphine complexes. Oxygen transfer from iodosylbenzene. J. Am. Chem. Soc..

[18] Jiang G., Liu Q., Guo C., George A. (2011). Biomimetic oxidation of hydrocarbons with air over metalloporphyrins. Biomimetic Based Applications.

[19] Meunier B. (1992). Metalloporphyrins as versatile catalysts for oxidation reactions and oxidative DNA cleavage. Chem. Rev..

[20] Chang C., Kuo M. (1979). Reaction of iron(III) porphyrins and iodosoxylene. The active oxene complex of cytochrome P-450. J. Am. Chem. Soc..

[21] Dolphin D., Traylor T.G., Xie L.Y. (1997). Polyhaloporphyrins: Unusual ligands for metals and metal-catalysed oxidations. Acc. Chem. Res..

[22] Traylor T.G., Tsuchiya S. (1987). Perhalogenated tetraphenylhemins: Stable catalysts of high turnover catalytic hydroxylations. Inorg. Chem..

[23] Haber J., Matachowski L., Pamin K., Poltowicz J. (2003). The effect of peripheral substituents in metalloporphyrins on their catalytic activity in Lyons system. J. Mol. Catal. A Chem..

[24] Srour H., le Maux P., Chevance S., Simonneaux G. (2013). Metal-catalysed asymmetric sulfoxidation, epoxidation and hydroxylation by hydrogen peroxide. Coord. Chem. Rev..

[25] Caron S., Dugger R.W., Ruggeri S.G., Ragan J.A., Brown Ripin D.H. (2006). Large-scale oxidations in the pharmaceutical industry. Chem. Rev..

[26] Lenoir D. (2006). Selective oxidation of organic compounds-sustainable catalytic reactions with oxygen and without transition metals?. Angew. Chem. Int. Ed. Engl..

[27] Denisov I.G., Makris T.M., Sligar S.G., Schlichting I. (2005). Structure and chemistry of cytochrome P450. Chem. Rev..

[28] Che C.-M., Kar-yan lo V., Zhou C.-Y., Huang J.-S. (2011). Selective functionalisation of saturated C–H bonds with metalloporphyrin catalysts. Chem. Soc. Rev..

[29] Guo C.-C., Liu X.-Q., Liu Q., Liu Y., Chu M.-F., Lin W.-Y. (2009). First industrial-scale biomimetic oxidation of hydrocarbon with air over metalloporphyrins as cytochrome P-450 monooxygenase model and its mechanistic studies. J. Porphyr. Phthalocyanines.

[30] Costas M. (2011). Selective C–H oxidation catalysed by metalloporphyrins. Coord. Chem. Rev..

[31] Nakagaki S., Ferreira G., Ucoski G., Dias de Freitas Castro K. (2013). Chemical reactions catalysed by metalloporphyrin-based metal-organic frameworks. Molecules.

[32] Akagah B., Lormier A.T., Fournet A., Figadère B. (2008). Oxidation of antiparasitic 2-substituted quinolines using metalloporphyrin catalysts: Scale-up of a biomimetic reaction for metabolite production of drug candidates. Org. Biomol. Chem..

[33] Che C.-M., Huang J.-S. (2009). Metalloporphyrin-based oxidation systems: From biomimetic reactions to application in organic synthesis. Chem. Commun..

[34] Groves J.T., Myers R.S. (1983). Catalytic asymmetric epoxidations with chiral iron porphyrins. J. Am. Chem. Soc..

[35] Rose E., Andrioletti B., Zrig S., Quelquejeu-Ethève M. (2005). Enantioselective epoxidation of olefins with chiral metalloporphyrin catalysts. Chem. Soc. Rev..

[36] Gopalaiah K. (2013). Chiral iron catalysts for asymmetric synthesis. Chem. Rev..

[37] Lane B.S., Burgess K. (2003). Metal-catalysed epoxidations of alkenes with hydrogen peroxide. Chem. Rev..

[38] De Faveri G., Ilyashenko G., Watkinson M. (2011). Recent advances in catalytic asymmetric epoxidation using the environmentally benign oxidant hydrogen peroxide and its derivatives. Chem. Soc. Rev..

[39] Adam W., Stegmann V.R., Saha-mo C.R. (1999). Regio- and diastereoselective epoxidation of chiral allylic alcohols catalysed by manganese (salen) and iron (porphyrin) complexes. J. Am. Chem. Soc..

[40] Rose E., Ren Q.Z., Andrioletti B. (2004). A Unique binaphthyl strapped iron-porphyrin catalyst for the enantioselective epoxidation of terminal olefins. Chem. Eur. J..

[41] Stephenson N.A., Bell A.T. (2007). Mechanistic insights into iron porphyrin-catalysed olefin epoxidation by hydrogen peroxide: Factors controlling activity and selectivity. J. Mol. Catal. A Chem..

[42] Vilain-Deshayes S., Robert A., Maillard P., Meunier B., Momenteau M. (1996). Enantioselective epoxidation of olefins by single-oxygen atom donors catalysed by manganese-glycoconjugated porphyrins. J. Mol. Catal. A Chem..

[43] Le Maux P., Srour H.F., Simonneaux G. (2012). Enantioselective water-soluble iron–porphyrin-catalysed epoxidation with aqueous hydrogen peroxide and hydroxylation with iodobenzene diacetate. Tetrahedron.

[44] Srour H., Le Maux P., Simonneaux G. (2012). Enantioselective manganese-porphyrin-catalysed epoxidation and C–H hydroxylation with hydrogen peroxide in water/methanol solutions. Inorg. Chem..

[45] Cunningham I.D., Danks T.N., Hay J.N., Hamerton I., Gunathilagan S. (2001). Evidence for parallel destructive, and competitive epoxidation and dismutation pathways in metalloporphyrin-catalysed alkene oxidation by hydrogen peroxide. Tetrahedron.

[46] Nam W., Jin S.W., Lim M.H., Ryu J.Y., Kim C. (2002). Anionic ligand effect on the nature of epoxidizing intermediates in iron porphyrin complex-catalysed epoxidation reactions. Inorg. Chem..

[47] Nam W., Oh S., Sun Y.J., Kim J., Kim W., Woo S.K. (2003). Factors affecting the catalytic epoxidation of olefins by iron porphyrin complexes and H_2_O_2_ in protic solvents. J. Org. Chem..

[48] Zhang J.L., Zhou H.B., Huang J.S., Che C.M. (2002). Dendritic ruthenium porphyrins: A new class of highly selective catalysts for alkene epoxidation and cyclopropanation. Chem. Eur. J..

[49] Oae S., Watanabe Y., Fujimori K. (1982). Biomimetic oxidation of organic sulfides with TPPFe(III)Cl/Imidazole/Hydrogen Peroxide. Tetrahedron Lett..

[50] Kowalski P., Mitka K., Ossowska K., Kolarska Z. (2005). Oxidation of sulfides to sulfoxides. Part 1: Oxidation using halogen derivatives. Tetrahedron.

[51] Legros J., Dehli J.R., Bolm C. (2005). Applications of catalytic asymmetric sulfide oxidations to the syntheses of biologically active sulfoxides. Adv. Synth. Catal..

[52] Wojaczyńska E., Wojaczyński J. (2010). Enantioselective synthesis of sulfoxides: 2000–2009. Chem. Rev..

[53] Baciocchi E., Gerini M.F., Lapi A. (2004). Synthesis of sulfoxides by the hydrogen peroxide induced oxidation of sulfides catalysed by iron tetrakis(pentafluorophenyl)porphyrin: Scope and chemoselectivity. J. Org. Chem..

[54] Kaczorowska K., Kolarska Z., Mitka K., Kowalski P. (2005). Oxidation of sulfides to sulfoxides. Part 2: Oxidation by hydrogen peroxide. Tetrahedron.

[55] Carreño M.C., Hernández-Torres G., Ribagorda M., Urbano A. (2009). Enantiopure sulfoxides: Recent applications in asymmetric synthesis. Chem. Commun..

[56] Le Maux P., Simonneaux G. (2011). First enantioselective iron-porphyrin-catalysed sulfide oxidation with aqueous hydrogen peroxide. Chem. Commun..

[57] Srour H., Jalkh J., le Maux P., Chevance S., Kobeissi M., Simonneaux G. (2013). Asymmetric oxidation of sulfides by hydrogen peroxide catalysed by chiral manganese porphyrins in water/methanol solution. J. Mol. Catal. A Chem..

[58] Mori T., Santa T., Higuchi T., Mashino T., Hirobe M. (1993). Oxygen activation by Iron(III)-porphyrin/NaBH_4_/Me_4_N·OH system as cytochrome P-450 Model. Oxygenation of olefin, *N*-dealkylation of tertiary amine, oxidation of sulfide, and oxidative cleavage of ether bond. Chem. Pharm. Bull..

[59] Ortiz De Montellano P.R. (2010). Hydrocarbon hydroxylation by cytochrome P450 enzymes. Chem. Rev..

[60] Vinhado F.S., Prado-Manso C.M.C., Sacco H.C., Iamamoto Y. (2001). Cationic manganese(III) porphyrins bound to a novel bis-functionalised silica as catalysts for hydrocarbons oxygenation by iodosylbenzene and hydrogen peroxide. J. Mol. Catal. A Chem..

[61] Campestrini S. (2001). Highly efficient cascade-oxygen-transfer from H_2_O_2_ to olefins mediated by halogenated carbonyl compounds and metalloporphyrins. J. Mol. Catal. A Chem..

[62] Shilov A.E., Shul G.B. (1997). Activation of C–H bonds by metal complexes. Chem. Rev..

[63] Groves J.T., Viski P. (1989). Asymmetric hydroxylation by a chiral iron porphyrin. J. Am. Chem. Soc..

[64] Groves J., Viski P. (1990). Asymmetric hydroxylation, epoxidation, and sulfoxidation catalysed by vaulted binaphthyl metalloporphyrins. J. Org. Chem..

[65] Gross Z., Ini S. (1999). Asymmetric catalysis by a chiral ruthenium porphyrin: Epoxidation, hydroxylation, and partial kinetic resolution of hydrocarbons. Org. Lett..

[66] Zhang R., Yu W.-Y., Che C.-M. (2005). Catalytic enantioselective oxidation of aromatic hydrocarbons with D_4_-symmetric chiral ruthenium porphyrin catalysts. Tetrahedron Asymmetry.

[67] De Freitas Silva G., da Silva D.C., Guimarães A.S., do Nascimento E., Rebouças J.S., de Araujo M.P., de Carvalho M.E.M.D., Idemori Y.M. (2007). Cyclohexane hydroxylation by iodosylbenzene and iodobenzene diacetate catalysed by a new β-octahalogenated Mn–porphyrin complex: The effect of meso-3-pyridyl substituents. J. Mol. Catal. A Chem..

[68] Ogawa S., Hosoi K., Iida T., Wakatsuki Y., Makino M., Fujimoto Y., Hofmann A.F. (2007). Osmiumporphyrin-catalysed oxyfunctionalization and isomerization of natural (5β)-bile acids with *tert*-butyl hydroperoxide. Eur. J. Org. Chem..

[69] Ogawa S., Wakatsuki Y., Makino M., Fujimoto Y., Yasukawa K., Kikuchi T., Ukiya M., Akihisa T., Iida T. (2010). Oxyfunctionalization of unactivated C-H bonds in triterpenoids with *tert*-butylhydroperoxide catalysed by *meso*-5,10,15,20-tetramesitylporphyrinate osmium(II) carbonyl complex. Chem. Phys. Lipids.

[70] Iida T., Ogawa S., Hosoi K., Makino M., Fujimoto Y., Goto T., Mano N., Goto J., Hofmann A.F. (2007). Regioselective oxyfunctionalization of unactivated carbons in steroids by a model of cytochrome P-450: Osmiumporphyrin complex/*tert*-butyl hydroperoxide system. J. Org. Chem..

[71] Ren Q.-G., Chen S.-Y., Zhou X.-T., Ji H.-B. (2010). Highly efficient controllable oxidation of alcohols to aldehydes and acids with sodium periodate catalysed by water-soluble metalloporphyrins as biomimetic catalyst. Bioorg. Med. Chem..

[72] Muzart J. (2003). Palladium-catalysed oxidation of primary and secondary alcohols. Tetrahedron.

[73] Ji H., Zhou X., Mukherjee A. (2010). Biomimetic homogeneous oxidation catalysed by metalloporphyrins with green oxidants. Biomimetic Learning from Nature.

[74] Adam W., Prikhodovski S., Roschmann K.J., Saha-Moller C.R. (2001). Oxidation of aryl-substituted allylic alcohols by an optically active Fe(III)(porph*) catalyst: Enantioselectivity, diastereoselectivity and chemoselectivity in the epoxide versus enone formation. Tetrahedron Asymmetry.

[75] Burri E., Öhm M., Daguenet C., Severin K. (2005). Site-isolated porphyrin catalysts in imprinted polymers. Chem. Eur. J..

[76] Han J.H., Yoo S., Seo S., Hong J., Kim K., Kim C. (2005). Biomimetic alcohol oxidations by an iron(III) porphyrin complex: Relevance to cytochrome P-450 catalytic oxidation and involvement of the two-state radical rebound mechanism. Dalton Trans..

[77] Rezaeifard A., Jafarpour M., Moghaddam G.K., Amini F. (2007). Cytochrome P-450 model reactions: Efficient and highly selective oxidation of alcohols with tetrabutylammonium peroxymonosulfate catalysed by Mn-porphyrins. Bioorg. Med. Chem..

[78] Du G., Keith Woo L. (2005). Alcohol oxidation with dioxygen mediated by oxotitanium porphyrin and related transition metal complexes. J. Porphyr. Phthalocyanines.

[79] Du G., Woo L.K. (2003). Synthesis, characterization, and reactivity of group 4 metalloporphyrin diolate complexes. Organometallics.

[80] Ji H.-B., Yuan Q.-L., Zhou X.-T., Pei L.-X., Wang L.-F. (2007). Highly efficient selective oxidation of alcohols to carbonyl compounds catalysed by ruthenium (III) *meso*-tetraphenylporphyrin chloride in the presence of molecular oxygen. Bioorg. Med. Chem. Lett..

[81] Rebelo S.L., Simões M.M., Neves M.G.P.M., Cavaleiro J.A. (2003). Oxidation of alkylaromatics with hydrogen peroxide catalysed by manganese(III) porphyrins in the presence of ammonium acetate. J. Mol. Catal. A Chem..

[82] Zhang J.L., Che C.M. (2005). Dichlororuthenium(IV) complex of *meso*-Tetrakis(2,6-dichlorophenyl) porphyrin: Active and robust catalyst for highly selective oxidation of arenes, unsaturated steroids, and electron-deficient alkenes by using 2,6-dichloropyridine *N*-oxide. Chem. Eur. J..

[83] Twarowski A.J. (1988). Porphyrin-sensitized formation of a polymer-bound endoperoxide in the solid state. J. Phys. Chem..

[84] Slavětínská L., Mosinger J., Kubát P. (2008). Supramolecular carriers of singlet oxygen: Photosensitized formation and thermal decomposition of endoperoxides in the presence of cyclodextrins. J. Photochem. Photobiol. A.

[85] Cossy J., Belotti D. (2001). Efficient synthesis of substituted quinoline-5,8-quinones from 8-hydroxyquinolines by photooxygenation. Tetrahedron Lett..

[86] Faustino M.A., Neves M.G., Cavaleiro J.A., Neumann M., Brauer H.D., Jori G. (2000). *meso*-Tetraphenylporphyrin dimer derivatives as potential photosensitizers in photodynamic therapy. Part 2. Photochem. Photobiol..

[87] Ogilby P.R. (2010). Singlet oxygen: There is indeed something new under the sun. Chem. Soc. Rev..

[88] Amarasekara A.S. (1999). A new synthesis of quinoline-5,8-quinone. Synth. Commun..

[89] Paddock V.L., Phipps R.J., Conde-Angulo A., Blanco-Martin A., Giró-Mañas C., Martin L.J., White A.J.P., Spivey A.C. (2011). (±)-*trans*,*cis*-4-Hydroxy-5,6-di-*O*-isopropylidenecyclohex-2-ene-1-one: Synthesis and facile dimerization to decahydrodibenzofurans. J. Org. Chem..

[90] Nicolaou K.C., Totokotsopoulos S., Giguère D., Sun Y., Sarlah D. (2011). Total synthesis of epicoccin G. J. Am. Chem. Soc..

[91] Murahashi S.-I. (1995). Synthetic aspects of metal-catalysed oxidations of amines and related reactions. Angew. Chem. Int. Ed. Engl..

[92] Matsumoto M., Kitano Y. (1996). Singlet Oxygenation of 1-aminomethyl-1-*tert*-butyl-2-methoxy-2-(3-methoxy-phenyl)ethylenes: Marked effect of allylic nitrogen on the reaction pathways and chemoselectivity. Tetrahedron Lett..

[93] Jiang G., Chen J., Huang J.-S., Che C.-M. (2009). Highly efficient oxidation of amines to imines by singlet oxygen and its application in Ugi-type reactions. Org. Lett..

[94] Nardello V., Hervé M., Alsters P.L., Aubry J.-M. (2002). “Dark” singlet oxygenation of hydrophobic substrates in environmentally friendly microemulsions. Adv. Synth. Catal..

[95] Coyle E.E., Joyce K., Nolan K., Oelgemöller M. (2010). Green photochemistry: The use of microemulsions as green media in photooxygenation reactions. Green Chem..

[96] Lu H., Zhang X.P. (2011). Catalytic C–H functionalization by metalloporphyrins: Recent developments and future directions. Chem. Soc. Rev..

[97] Song C.E., Lee S.G. (2002). Supported chiral catalysts on inorganic materials. Chem. Rev..

[98] Leadbeater N.E., Marco M. (2002). Preparation of polymer-supported ligands and metal complexes for use in catalysis. Chem. Rev..

[99] Ribeiro S.M., Serra A.C., Rocha Gonsalves A.M.D. (2010). Covalently immobilized porphyrins on silica modified structures as photooxidation catalysts. J. Mol. Catal. A Chem..

[100] Benaglia M., Danelli T., Fabris F., Sperandio D., Pozzi G. (2002). Poly(ethylene glycol)-supported tetrahydroxyphenyl porphyrin: A convenient, recyclable catalyst for photooxidation reactions. Org. Lett..

[101] Zhang J.-L., Huang J.-S., Che C.-M. (2006). Oxidation chemistry of poly(ethylene glycol)-supported carbonylruthenium(II) and dioxoruthenium(VI)meso-Tetrakis(pentafluorophenyl)porphyrin. Chem. Eur. J..

[102] Shiragami T., Makise R., Inokuchi Y., Matsumoto J., Inoue H., Yasuda M. (2004). Efficient photocatalytic oxidation of cycloalkenes by dihydroxo(tetraphenylporphyrinato)antimony supported on silica gel under visible light irradiation. Chem. Lett..

[103] Azenha E.G., Serra A.C., Pineiro M., Pereira M.M., Seixas de Melo J., Arnaut L.G., Formosinho S.J., Rocha Gonsalves A.M.D. (2002). Heavy-atom effects on metalloporphyrins and polyhalogenated porphyrins. Chem. Phys..

[104] Ribeiro S., Serra A.C., Gonsalves A.R. (2013). Efficient solar photooxygenation with supported porphyrins as catalysts. ChemCatChem.

[105] Wegner J., Ceylan S., Kirschning A. (2012). Flow chemistry—A key enabling technology for (multistep) organic synthesis. Adv. Synth. Catal..

[106] Wiles C., Watts P. (2012). Continuous flow reactors: A perspective. Green Chem..

[107] Oelgemöller M., Shvydkiv O. (2011). Recent advances in microflow photochemistry. Molecules.

[108] Griesbeck A.G., El-Idreesy T.T. (2005). Solvent-free photooxygenation of 5-methoxyoxazoles in polystyrene nanocontainers doped with tetrastyrylporphyrine and protoporphyrine-IX. Photochem. Photobiol. Sci..

[109] Bourne R.A., Han X., Poliakoff M., George M.W. (2009). Cleaner continuous photo-oxidation using singlet oxygen in supercritical carbon dioxide. Angew. Chem. Int. Ed..

[110] Bourne R.A., Han X., Chapman A.O., Arrowsmith N.J., Kawanami H., Poliakoff M., George M.W. (2008). Homogeneous photochemical oxidation via singlet O_2_ in supercritical CO_2_. Chem. Commun..

[111] Amara Z., Bellamy J.F.B., Horvath R., Miller S.J., Beeby A., Burgard A., Rossen K., Poliakoff M., George M.W. (2015). Applying green chemistry to the photochemical route to artemisinin. Nat. Chem..

[112] Han X., Bourne R.A., Poliakoff M., George M.W. (2011). Immobilised photosensitisers for continuous flow reactions of singlet oxygen in supercritical carbon dioxide. Chem. Sci..

[113] Loponov K.N., Lopes J., Barlog M., Astrova E.V., Malkov A.V., Lapkin A.A. (2014). Optimization of a scalable photochemical reactor for reactions with singlet oxygen. Org. Process Res. Dev..

[114] Lévesque F., Seeberger P.H. (2011). Highly efficient continuous flow reactions using singlet oxygen as a “Green” reagent. Org. Lett..

[115] Lévesque F., Seeberger P.H. (2012). Continuous-flow synthesis of the anti-malaria drug artemisinin. Angew. Chem. Int. Ed..

[116] Turconi J., Griolet F., Guevel R., Oddon G., Villa R., Geatti A., Hvala M., Rossen K., Göller R., Burgard A. (2014). Semisynthetic artemisinin, the chemical path to industrial production. Org. Process Res. Dev..

[117] De Oliveira K.T., Miller L.Z., McQuade D.T. (2016). Exploiting photooxygenations mediated by porphyrinoid photocatalysts under continuous flow conditions. RSC Adv..

[118] Momo P.B., Bellete B.S., Brocksom T.J., de Souza R.O.M.A., de Oliveira K.T. (2015). Exploiting novel process windows for the synthesis of meso-substituted porphyrins under continuous flow conditions. RSC Adv..

